# Understanding medical students’ continuance intention to use VR-based learning systems: an integrated model approach

**DOI:** 10.3389/fmed.2025.1708428

**Published:** 2026-01-20

**Authors:** Yanhong Zhang, Jingcheng Liu, Yiling Zhenghuang, Xiaoning Lu, Xinxin Zhang

**Affiliations:** 1 Shaanxi Normal University, Xi’an, Shaanxi, China; 2 Silla University, Sasang-gu, Busan, Republic of Korea; 3 Jiangxi Province Jingdezhen City Sports School, Jingdezhen, China; 4 Guangxi Normal University, Guangxi, China

**Keywords:** continued intention, integrated model, medical students, structural equation modeling, VR-based learning systems

## Abstract

As Virtual Reality (VR) technology advances, VR-based learning systems offer medical students immersive, repeatable, and risk-free simulation environments, which are crucial for clinical skill development. Continued Intention (CI) to use these systems is a key determinant of their long-term educational impact. This study investigates the factors influencing medical students’ CI by proposing an integrated research model grounded in the Unified Theory of Acceptance and Use of Technology and continuance theory. The model posits that System Characteristics (SC), Social Influence (SI), and Facilitating Conditions (FC) influence CI indirectly through the mediating roles of Perceived Ease of Use (PE) and Perceived Usefulness (PU). Survey data were collected from 258 medical students at Chinese universities with prior experience with VR learning systems and analyzed using Structural Equation Modeling. The results confirm that SC, SI, and FC exert no direct effects on CI but are fully mediated by PE and PU. Specifically, PE mediated the effects of FC and SI on CI, while PU mediated the impact of SC and SI on CI. Based on these identified pathways (e.g., SC→PU→CI; SI→PE→CI), we provide targeted recommendations: a) Enhancing system design and content relevance to improve perceived usefulness directly; b) Leveraging social proof and learning communities to strengthen perceptions of ease of use and usefulness; and c) Optimizing technical and instructional support to reduce usage barriers and foster positive user experience. This study offers theoretical insights into the post-adoption behavior of VR systems and practical guidance for promoting their sustained integration into medical curricula.

## Introduction

Medical education faces persistent challenges, including limited opportunities for practical training and the high-stakes nature of clinical environments where patient safety restricts student involvement (1, 2). Virtual reality (VR)-based learning systems have emerged as a transformative pedagogical tool, creating immersive, repeatable, and risk-free simulation environments that can bridge the gap between theory and practice (3–5).

Research on the implementation of VR in medical education is growing. Numerous studies have employed technology acceptance theories, such as the Technology Acceptance Model (TAM) and the Unified Theory of Acceptance and Use of Technology (UTAUT), to understand initial adoption by students and educators (6–9). However, the long-term success and educational return on investment of these systems depend not only on initial adoption, but also on continuance intention (CI)—defined as the user’s intention to continue using an information system after its initial adoption (10). Research specifically examining the post-adoption continuance intention to use VR systems among medical students, and the mechanisms by which external factors shape this intention, remains underdeveloped. Understanding these drivers is critical, as not all implemented VR systems achieve sustained engagement, which ultimately affects their pedagogical value and institutional impact (2, 6).

To address this gap, this study develops an integrated research model. We draw on the UTAUT and continuance theory to examine how key external factors—System Characteristics (SC), Social Influence (SI), and Facilitating Conditions (FC)—influence medical students’ CI. The model posits that these external factors exert their influence indirectly through the mediating roles of Perceived Ease of Use (PE) and Perceived Usefulness (PU), which are established core determinants of both initial and continued usage.

This study is situated within the context of Chinese higher medical education. Medical students are the focal population, as they are the primary end users of VR simulation training. A critical methodological prerequisite is that all participants have prior hands-on experience with a VR learning system, thereby ensuring the validity of measuring post-adoption continuance intention. Using survey data and Structural Equation Modeling (SEM) (11, 12), this study aims to elucidate the specific mediating pathways (e.g., SC→PU→CI) that underlie sustained usage. The findings are intended to provide theoretical insights into the post-adoption phase and evidence-based, practical recommendations for the design and implementation strategies that can support the sustained and effective integration of VR-based learning systems in medical curricula.

## Theoretical research and analysis

### Literature review

#### Virtual reality in medical education

VR technology uses computer systems to generate three-dimensional images or environments. Prior studies indicate that the use of VR technology to create virtual environments for simulation-based training constitutes a notable and promising educational strategy, representing a significant advancement in educational technology development (13–15). VR teaching methods can simulate processes in the physical world and forecast natural and social phenomena that are not feasible to replicate or experiment with in the real world (3, 7, 16). VR teaching systems are widely used in medical education, primarily for developing technical competencies (17–19). VR teaching systems are used for surgical skills training and to enhance the ability to visualize three-dimensional anatomical structures (16, 20). Hammouda et al. assessed the efficacy of a VR human anatomy simulation training program for undergraduate students in Tunisia (3). Aydin et al. conducted a crossover study that concluded VR teaching systems can aid healthcare professionals in training for neonatal resuscitation programs (21). Conversely, VR teaching systems may be used to teach soft skills, such as empathy and communication. Jans et al. conducted a comprehensive review revealing that research on VR applications indicates its potential to enhance critical thinking, clinical reasoning, clinical judgment, and clinical decision-making skills in undergraduate nursing students (22). Bracq et al. conducted a systematic review and meta-analysis, revealing that the non-technical skills emphasized in VR teaching systems mainly encompass teamwork, communication, and situational awareness (23). VR technology has considerable potential to enhance medical education (24). VR-based instructional systems can significantly aid future physicians in managing complex emergency medical situations, thus promoting the incorporation of these technologies into medical curricula (25).

#### Theoretical foundations: from initial adoption to continuance intention

The TAM, introduced by Davis (26), is a foundational theory for explaining initial adoption intention toward information systems (26). It posits that PE and PU are key determinants of an individual’s intention to use a new technology (26). To provide a more comprehensive understanding of technology acceptance, Venkatesh et al. synthesized eight competing models and proposed the UTAUT (27). UTAUT identifies four core direct determinants of usage intention: Performance Expectancy (similar to PU), Effort Expectancy (similar to PE), SI, and FC. Furthermore, UTAUT specifies four moderators (gender, age, experience, and voluntariness of use). The constructs of SI and FC, as defined in our study, are drawn directly from the UTAUT framework.

It is critical to note that both TAM and UTAUT were developed primarily to predict initial adoption behavior. However, the long-term success of a technology depends on users’ CI—the decision to continue using a system after its initial adoption (26, 28). To explain this post-adoption behavior, Bhattacherjee’s Expectation-Confirmation Model (ECM) of IS Continuance is the predominant theory (10). The ECM posits that continuance intention is primarily determined by user satisfaction and post-adoption perceived usefulness, both of which are shaped by the confirmation of pre-usage expectations (29).

#### Synthesis and positioning of the present study

In information systems research, it is recognized that cognitive and social factors that influence initial adoption often remain relevant in the post-adoption phase. Consequently, scholars have successfully integrated constructs from adoption theories (like UTAUT/TAM) with the continuance paradigm (ECM) to investigate sustained usage (2). This integrative approach allows for a nuanced understanding of how ongoing perceptions and external conditions drive continued use.

Following this integrative approach, the present study develops a research model where continuance intention to use serves as the ultimate dependent variable, consistent with ECM. To explain CI, the model incorporates key UTAUT constructs—namely, SI and FC—alongside a construct capturing SC. These external variables are hypothesized to shape users’ core cognitive evaluations, namely PE and PU (from TAM), which in turn are positioned as direct and mediating antecedents of CI. This model thus examines the indirect pathways through which external factors influence the sustained use of VR-based learning systems among medical students.

### Research hypotheses

Building on the integrated theoretical framework outlined, this study proposes a research model to explain medical students’ intention to continue using VR-based learning systems. The model positions CI as the dependent variable, consistent with the post-adoption paradigm. Drawing on the UTAUT framework and prior continuance research, three external constructs—SC, SI, and FC—are introduced as antecedents (30). These external factors are hypothesized to influence CI indirectly by shaping users’ core cognitive perceptions: PE and PU, which are established as direct predictors of both initial and continued usage intention (10, 27). The specific research hypotheses are developed as follows.

#### The influence of SC

SC refers to the functional and interactive attributes of the VR learning system, such as interface design, logical workflow, and interactivity (31, 32). High-quality system design is expected to influence users’ perceptions of its usability and utility positively. Prior research in educational technology continuance has shown that system quality is a significant antecedent to PE and PU (2, 32). Therefore, we hypothesize:

*H1*: SC positively influences PE regarding the VR learning system.

*H2*: SC positively influences PU regarding the VR learning system.

#### The influence of SI

SI refers to the degree to which a medical student perceives that essential others (e.g., peers, instructors, the broader academic community) believe they should use the system (33, 34). In the context of post-adoption behavior, social norms and peer recommendations can reinforce and validate one’s ongoing perceptions of a technology. Studies on the continuance use of e-learning systems have confirmed the role of SI in shaping PU and PE (34, 35). Therefore, we hypothesize:

*H3*: SI positively influences PE regarding the VR learning system.

*H4*: SI positively influences PU regarding the VR learning system.

#### The influence of FC

FC refers to the degree to which a medical student believes that organizational and technical infrastructure exists to support the use of the system (e.g., accessible guidance, reliable technical support, adequate resources) (36). In a continuance context, strong facilitating conditions can reduce post-adoption effort and reinforce the perception that continued use is straightforward. Research in mobile learning continuance has linked FC to PE (9, 33, 36). Therefore, we hypothesize:

*H5*: FC positively influences PE regarding the VR learning system.

*H6*: FC positively influences PU regarding the VR learning system.

#### The core relationships from the integrated model

The integrated model retains the core relationships posited by TAM but applies them to predict continuance intention. Specifically, PE is expected to directly affect both PU and CI, whereas PU is expected to directly affect CI. These relationships have been validated in numerous continuance studies that integrate TAM constructs (1, 10, 32). Therefore, we hypothesize:

*H7*: PE positively influences PU.

*H8*: PE positively influences CI.

*H9*: PU positively influences CI.

**FIGURE 1:**
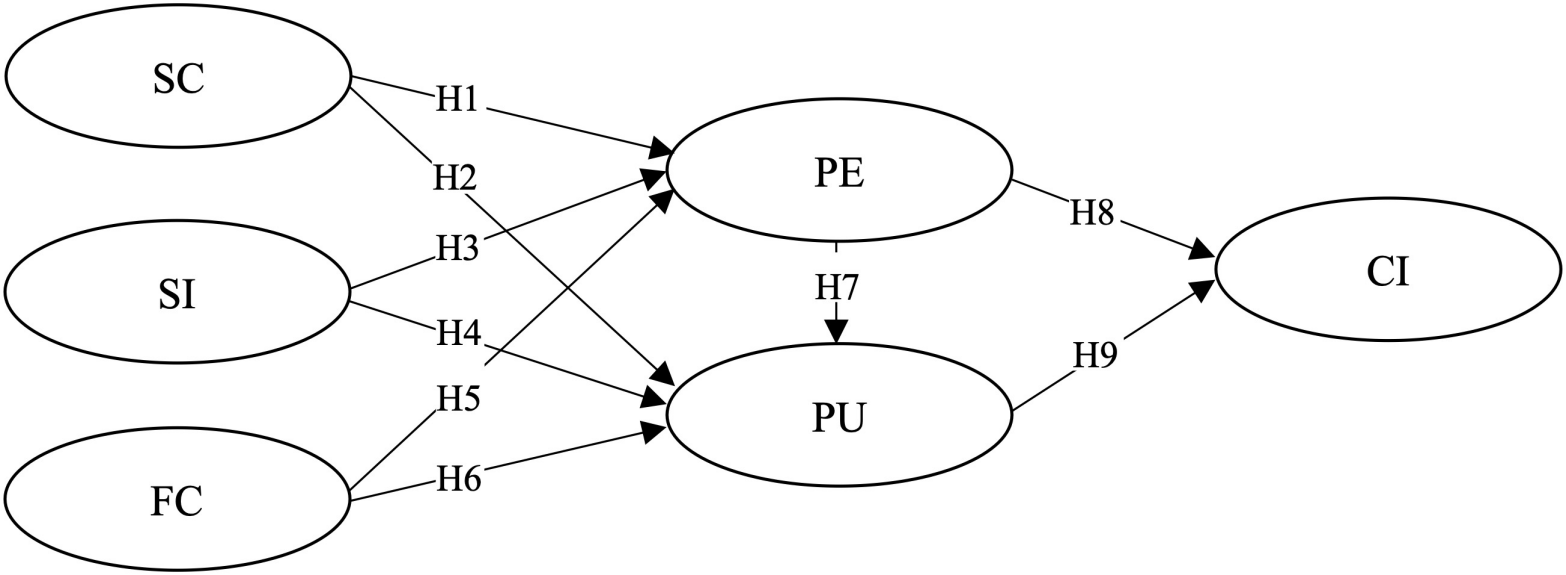
Research framework. H1–H9 refer to the nine hypotheses.

The resulting research model integrating these hypotheses is presented in Figure 1.

## Materials and methods

### Research methods

This study employed a cross-sectional survey design. Data analysis was performed following a two-step SEM approach using SPSS 26.0 and AMOS 26.0. This analytical strategy allows simultaneous testing of the measurement model (relationships between indicators and latent constructs) and the structural model (hypothesized paths between constructs), which is well-suited for assessing the plausibility of our pre-specified theoretical framework. It is important to note that, while SEM tests hypothesized causal relationships, the cross-sectional nature of our data supports interpreting these findings as robust associative evidence for the proposed model rather than definitive proof of causality.

### Questionnaire design

Data were collected via a structured questionnaire. To ensure the validity of measuring continuance intention to use, participants were required to confirm they had used a VR-based learning system at least once in the past month. The questionnaire comprised: a) demographic items and b) measurement scales for all model constructs, adapted from established literature (Table 1). All items used a seven-point Likert scale (1 = Strongly Disagree, 7 = Strongly Agree). To enhance content validity and clarity, a pilot study was conducted with 50 eligible medical students. Feedback led to minor wording improvements, confirming the instrument’s comprehensibility.

**TABLE 1:** List of questions.

Construct	Code	Variable measurement content	Source
SC	SC1	The VR teaching system should feature an intuitive interface and clear navigation settings.	Dehghani and Mashhadi (14); Samadzad et al. (15)
	SC2	All functions of the VR teaching system should be logically designed for user-friendly operation and utilization.	
	SC3	The VR teaching system should incorporate high-quality interactive design capabilities.	
SI	SI1	Due to promotional coverage across various media channels, I would be inclined to experience and utilize the VR teaching system.	Cioc et al. (16); Argan et al. (17)
	SI2	Teachers’ recommendations would influence my decision to experience and utilize the VR teaching system.	
	SI3	Those around me endorse the VR teaching system.	
FC	FC1	I find it straightforward to use the VR teaching system in my daily routine.	Rani et al. (18); Ebadi and Raygan (19)
	FC2	The VR teaching system provides guidance to assist me in experiencing and utilizing it.	
	FC3	The VR teaching system provides relevant services to resolve issues encountered during use.	
PE	PE1	Learning to use this VR teaching system was straightforward for me.	Davis (7)
	PE2	I can control the VR teaching system to accomplish my desired tasks.	
	PE3	I found this VR teaching system remarkably user-friendly.	
PU	PU1	I believe using this VR teaching system aids my learning.	Davis (7)
	PU2	I found learning with this VR teaching system highly beneficial.	
	PU3	I gained significant insights from using this VR teaching system.	
CI	CI1	I would use this system again for learning if possible.	Davis (7); Mailizar et al. (13)
	CI2	I am willing to use this VR teaching system once more.	
	CI3	Compared to other VR learning formats, I prefer VR teaching system.	

### Data collection and screening

The online survey was distributed to medical students at Chinese universities. To mitigate response bias, the introduction emphasized anonymity and academic-use only. Two instructed attention-check items (e.g., “Please select ‘Agree’ for this statement”) were embedded to identify inattentive respondents. Data cleaning involved a multi-step protocol: (a) Removal of 13 responses with completion times < 60 seconds. (b) Examination of missing data. The rate was minimal (< 0.5% per variable), and Little’s MCAR test was non-significant (χ^2^ = 15.32, *p* > 0.05), supporting the use of Full Information Maximum Likelihood (FIML) estimation in SEM for handling missingness. This yielded 258 valid responses for analysis. The final sample characteristics are in Table 2. The restriction to recent VR users, while essential for measuring post-adoption continuance, is acknowledged as a factor affecting generalizability to novice populations.

**TABLE 2:** Demographics of respondents (*n* = 258).

Category		Number	Percent (%)
Age	18 years old and under	52	20.16
	19 years old	81	31.40
	20 years old	50	19.38
	21 years old	46	17.83
	22 years old and over	29	11.24
Gender	Male	121	46.90
	Female	137	53.10
Year group	Year 1	67	25.97
	Year 2	63	24.42
	Year 3	79	30.62
	Year 4	49	18.99
Level of familiarity with VR teaching systems	Not at all familiar	23	8.91
	Somewhat familiar	46	17.83
	Fairly familiar	87	33.72
	Quite familiar	53	20.54
	Very familiar	49	18.99

To focus on post-adoption behavior, all participants confirmed prior experience with VR learning systems. Other potential covariates (e.g., detailed technology use history, academic performance) were not collected in this study and are acknowledged as a limitation for future research. The high valid response rate should be interpreted considering the potential for self-selection bias common in online surveys.

### Data analysis procedures

Before SEM, data were screened using SPSS. Univariate normality was assessed using skewness and kurtosis (all within ± 2 and ± 7). Multicollinearity was examined by calculating Variance Inflation Factors (VIFs) for all predictor constructs in a regression framework; all VIFs were below 2.0, indicating no issue. Multivariate outliers were assessed using Mahalanobis distance (*p* < 0.001), and none were found.

We evaluated reliability and validity. Convergent validity was established by requiring: (a) all indicator factor loadings > 0.70 and statistically significant, (b) Composite Reliability (CR) > 0.80, and (c) Average Variance Extracted (AVE) > 0.50 (37). Discriminant validity was confirmed using the Fornell-Larcker criterion (AVE square root > inter-construct correlations).

After confirming a satisfactory measurement model, the structural model (Figure 1) was evaluated. Model fit was assessed using χ^2^/df, Comparative Fit Index (CFI), Tucker-Lewis Index (TLI), Root Mean Square Error of Approximation (RMSEA), and Standardized Root Mean Square Residual (SRMR) (38). No *post hoc* model modifications were performed to maintain theoretical integrity. Path coefficients were examined to test hypotheses H1-H9. To test the proposed mediating effects (H1-H6), a bias-corrected bootstrap procedure with 5,000 resamples was used to estimate 95% confidence intervals for indirect effects.

## Results

### Reliability and validity

The measurement model demonstrated strong psychometric properties. As shown in Table 3, all factor loadings were significant and exceeded 0.70 (range: 0.75–0.94). All constructs exhibited high internal consistency, with CR values ranging from 0.86 to 0.93. All AVE values exceeded 0.50 (range: 0.62–0.77), confirming convergent validity. Discriminant validity was established, as the square root of each construct’s AVE (diagonal in Table 4) was greater than its correlations with other constructs.

**TABLE 3:** Measurement model (convergent validity and reliability).

Construct	Item	Significance estimate				Topic reliability		AVE	CR
5pt.		Unstd. Factor loading	SE.	*z*-value	*P*-value	Std. factor loading	SMC		
SC	SC1	1.006	0.061	16.460	***	0.865	0.748	0.740	0.895
	SC2	1.008	0.061	16.500	***	0.867	0.752		
	SC3	1.000				0.848	0.719		
SI	SI1	1.184	0.066	17.926	***	0.931	0.867	0.781	0.914
	SI2	1.041	0.06	17.449	***	0.903	0.815		
	SI3	1.000				0.813	0.661		
FC	FC1	0.782	0.038	20.527	***	0.854	0.729	0.824	0.933
	FC2	1.012	0.04	25.465	***	0.944	0.891		
	FC3	1.000				0.922	0.850		
PE	PE1	0.899	0.048	18.732	***	0.864	0.746	0.787	0.917
	PE2	1.001	0.049	20.560	***	0.916	0.839		
	PE3	1.000				0.881	0.776		
PU	PU1	1.000				0.87	0.757	0.779	0.913
	PU2	1.044	0.056	18.548	***	0.878	0.771		
	PU3	1.098	0.057	19.178	***	0.899	0.808		
CI	CI1	1.060	0.058	18.198	***	0.913	0.834	0.791	0.919
	CI2	1.113	0.060	18.541	***	0.929	0.863		
	CI3	1.000				0.822	0.676		

SE, standard error; CR, critical ratio; P, *p*-value; SMC, squared multiple correlations.

***p < 0.001.

**TABLE 4:** Analysis of discriminant validity (Fornell-Larcker Criterion).

Construct	Convergent validity	Discriminant validity					
	AVE	PU	CI	PE	FC	SI	SC
PU	0.779	**0.883**					
CI	0.791	0.472	**0.889**				
PE	0.787	0.423	0.546	**0.887**			
FC	0.824	0.370	0.506	0.440	**0.908**		
SI	0.781	0.436	0.545	0.376	0.354	**0.884**	
SC	0.740	0.382	0.497	0.342	0.282	0.301	**0.860**

The correlations among constructs, such as that between PE and PU (0.546), are consistent with theoretical expectations in TAM/UTAUT-based models, where ease of use is a postulated antecedent of usefulness. The Fornell-Larcker criterion (diagonal values greater than off-diagonal correlations) was satisfied, supporting discriminant validity. Values in bold are the square roots of AVE.

### Model fit and hypothesis testing

The structural model demonstrated a good fit to the data (χ^2^/df = 2.55, CFI = 0.95, TLI = 0.938, RMSEA = 0.078, SRMR = 0.0435), with all indices meeting established thresholds for acceptability. This supports the plausibility of the hypothesized model for testing the proposed relationships.

**FIGURE 2:**
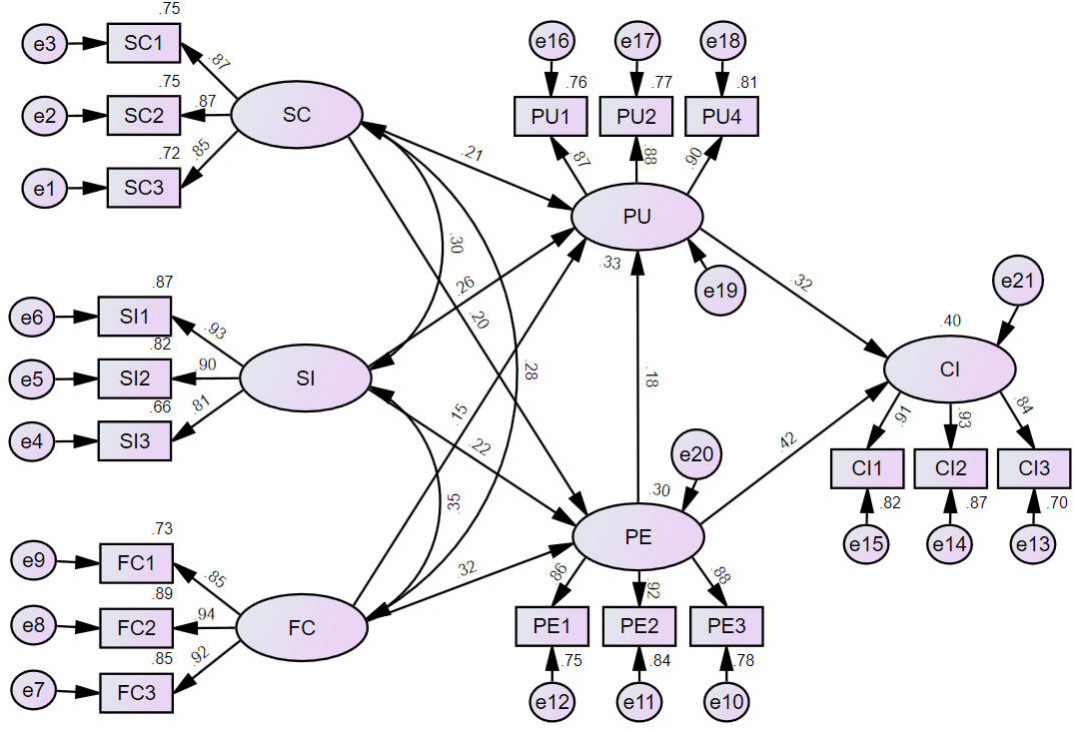
Path diagram. Values on the straight arrows between variables represent the standardized path coefficients.

As presented in Figure 2 and Table 5, all nine research hypotheses (H1–H9) were supported by significant path coefficients. Specifically, the external constructs exerted significant influences on the core mediators: SC, SI, and FC all positively affected both PE (H1, H3, H5: β = 0.201, 0.217, 0.318, respectively, all *p* < 0.01) and PU (H2, H4, H6: β = 0.207, 0.261, 0.149, respectively, all *p* < 0.05). In turn, PE positively influenced both PU (H7: β = 0.182, p = 0.010) and CI (H8: β = 0.430, *p* < 0.001). PU also exhibited a strong direct effect on CI (H9: β = 0.311, *p* < 0.001).

**TABLE 5:** Model fitting index.

Index	Model indicator values	Standard	Conclusion	Source
CMIN	313.601	The smaller, the better	Acceptable	
DF	123.000	The smaller, the better	Acceptable	
CMIN/DF	2.550	< 3	Good fit	
GFI	0.882	> 0.8 Acceptable; >0.9 Good fit	Acceptable	Bagozzi and Yi (24)
AGFI	0.837	> 0.8 Acceptable; >0.9 Good fit	Acceptable	
CFI	0.950	> 0.9	Good fit	Bagozzi and Yi (24)
TLI(NNFI)	0.938	> 0.9	Good fit	
RMSEA	0.078	< 0.08	Good fit	Bagozzi and Yi (24)
SRMR	0.0435	< 0.08	Good fit	Hu and Bentler (25)

CMIN, Chi-Square Minimum Fit Function; DF, Degrees of Freedom; CMIN/DF, Chi-Square to Degrees of Freedom ratio; GFI, Goodness of Fit Index; AGFI, Adjusted Goodness of Fit Index; CFI, Comparative Fit Index; TLI, Tucker-Lewis Index; NNFI, Non-Normed Fit Index; RMSEA, Root Mean Square Error of Approximation; SRMR, Standardized Root Mean Square Residual. No *post hoc* modifications were applied to preserve the theoretical integrity of the hypothesized model. The largest modification index (MI = 15.728) suggested a covariance between measurement error terms, which did not correspond to a theoretically justifiable relationship.

This pattern of results provides comprehensive support for the integrated model. Crucially, the significant paths from all three external factors (SC, SI, FC) to the mediators (PE and PU), coupled with the strong, significant paths from the mediators to CI—and the absence of any specified direct paths from the external factors to CI—empirically confirm the proposed fully mediated relationship structure. Perceived Ease of Use emerged as the most influential direct determinant of continuance intention.

### Mediation analysis

To test the proposed mediation mechanisms, a bootstrap analysis was performed. The results (Table 6) confirmed that all indirect paths from the external constructs to CI were statistically significant, while any hypothesized direct paths were not specified in the model and were found to be non-significant upon testing, collectively supporting a model of full mediation.

**TABLE 6:** Hypothesizes testing.

Hypothesis	Relationship	Unstd.	S.E.	C.R.	P	Std.(β)	Results	*R* ^2^
H1	SC→PE	0.232	0.074	3.126	0.002	0.201	Supported	0.3
H3	SI→PE	0.255	0.077	3.320	< 0.001	0.217	Supported	
H5	FC→PE	0.321	0.065	4.933	< 0.001	0.318	Supported	
H2	SC→PU	0.202	0.064	3.161	0.002	0.207	Supported	0.332
H4	SI→PU	0.261	0.067	3.912	< 0.001	0.261	Supported	
H6	FC→PU	0.128	0.057	2.235	0.025	0.149	Supported	
H7	PE→PU	0.154	0.060	2.572	0.010	0.182	Supported	
H8	PE→CI	0.372	0.057	6.580	< 0.001	0.430	Supported	0.395
H9	PU→CI	0.317	0.065	4.885	< 0.001	0.311	Supported	

A detailed examination of the specific indirect pathways (Table 7) reveals how each external factor operates. First, both SC and SI influence CI by enhancing PU (SC→PU→CI: β = 0.064; SI→PU→CI: β = 0.081). This indicates that a well-designed VR system and positive social norms promote sustained use primarily by convincing medical students of the system’s learning value. Second, SI and, more distinctly, FC influence CI by improving PE (SI→PE→CI: β = 0.093; FC→PE→CI: β = 0.137). This suggests that a supportive institutional environment and peer endorsements foster continued engagement by reducing the perceived effort required to use the technology.

**TABLE 7:** Mediation analysis.

Path	Effect type	SE	S.E.	Bootstrapping				Two-tailed significance	Std.(β)
				Bias-corrected 95% CI		Percentile 95% CI			
				Lower	Upper	Lower	Upper		
SC→PU→CI	Total effect	0.132	0.054	0.025	0.239	0.029	0.242	0.010	–
	Direct effect	0.000	0.000	0.000	0.000	0.000	0.000	>0.050	–
	Indirect effect	0.129	0.054	0.025	0.239	0.029	0.242	0.010	0.064
SI→PU→CI	Total effect	0.165	0.077	0.013	0.313	0.022	0.311	0.034	–
	Direct effect	0.000	0.000	0.000	0.000	0.000	0.000	>0.050	–
	Indirect effect	0.158	0.077	0.013	0.313	0.010	0.311	0.0340	0.081
FC→PU→CI	Total effect	0.102	0.074	-0.008	0.480	-0.014	0.274	>0.050	–
	Direct effect	0.000	0.000	0.000	0.000	0.000	0.000	>0.050	–
	Indirect effect	0.113	0.074	-0.008	0.274	-0.007	0.274	>0.050	0.046
PE→PU→CI	Total effect	0.545	0.063	0.410	0.655	0.412	0.656	<0.001	–
	Direct effect	0.417	0.083	0.237	0.564	0.231	0.561	<0.001	–
	Indirect effect	0.128	0.046	0.059	0.238	0.058	0.236	<0.001	0.057
SC→PE→CI	Total effect	0.118	0.085	-0.044	0.290	-0.058	0.272	>0.050	–
	Direct effect	0.000	0.000	0.000	0.000	0.000	0.000	>0.050	–
	Indirect effect	0.118	0.085	-0.044	0.290	-0.058	0.272	>0.050	0.086
SI→PE→CI	Total effect	0.128	0.049	0.034	0.225	0.039	0.229	0.009	–
	Direct effect	0.000	0.000	0.000	0.000	0.000	0.000	>0.050	–
	Indirect effect	0.128	0.049	0.034	0.225	0.039	0.229	0.009	0.093
FC→PE→CI	Total effect	0.183	0.052	0.083	0.288	0.090	0.297	0.001	–
	Direct effect	0.000	0.000	0.000	0.000	0.000	0.000	>0.050	–
	Indirect effect	0.183	0.052	0.083	0.288	0.090	0.297	0.001	0.137

The standardized indirect effect (β) was calculated by multiplying the standardized path coefficients from Table 6. It represents the effect size of the mediation. For paths where the bootstrap confidence interval includes zero (FC→PU→CI, SC→PE→CI), the indirect effect is not statistically significant.

The standardized indirect effects (β) ranged from 0.057 to 0.137 for the significant specific indirect paths. Following Cohen’s guidelines, these represent small to moderate effect sizes. Notably, the path from Facilitating Conditions to CI via PE (β = 0.137) was the strongest, highlighting the critical role of institutional support in lowering usage barriers. In summary, the mediation analysis provides robust evidence that the influence of SC, SI, and FC on CI is fully mediated by PE and PU. The distinct pathways identified clarify the cognitive mechanisms through which different external factors sustain engagement with VR learning systems.

## Discussion

### Interpretation of key findings

This study proposed and tested an integrated model to explain medical students’ CI to use VR-based learning systems. The results strongly support the model’s central premise: external factors—SC, SI, and FC—influence CI indirectly, with PE and PU serving as full mediators. This pattern underscores that for post-adoption engagement, objective features and social-environmental factors must positively shape users’ core cognitive evaluations to sustain their intention to continue using the technology.

The significant pathways from PE and PU to CI reaffirm their foundational role not only in initial adoption but also in continuance (26–28, 39, 40). The finding that PE affects CI both directly and indirectly (via PU) suggests that reducing operational complexity is a critical first step that also amplifies the system’s perceived value (41, 42). Conversely, PU’s direct effect on CI highlights that the sustained motivation to use the system is ultimately driven by a clear appraisal of its benefits for learning (43).

### Comparison with prior research

The findings of this study extend and refine existing knowledge on technology continuance in educational contexts. First, the confirmation of a fully mediated model, where SC, SI, and FC influence CI only through PE and PU, aligns with and strengthens a growing body of research advocating for integrated models in post-adoption studies (2, 31). This pattern suggests that, after initial adoption, objective and social-environmental factors must translate into positive user cognitions to sustain behavior, a nuance that pure adoption models like UTAUT do not fully capture.

Second, the significant SC→PU path (H2) resonates with prior work highlighting system quality as a cornerstone of perceived usefulness in e-learning (31). For medical VR, this implies that beyond technological immersion, clinical fidelity and pedagogical relevance are paramount for sustaining use (25). Conversely, the non-significant SC→PE→CI pathway, alongside the significant FC→PE path (H5), suggests that for these users, ease of use is less about intrinsic system design elegance and more about external support structures. This finding partially contrasts with some e-learning studies and highlights the unique technical and cognitive demands of VR, making institutional support crucial.

Third, the dual role of SI in enhancing both PU (H4) and PE (H3) underscores its enduring power in the post-adoption stage. This extends the UTAUT framework into continuance contexts, confirming that recommendations from peers and instructors continue to shape usefulness and ease-of-use evaluations over time (26, 27). In the collective learning environment of medical education, SI may be a particularly potent lever for sustaining engagement.

Finally, the strong direct effect of PE on CI (H8), even stronger than that of PU on CI (H9), offers a critical insight. While PU is often the dominant predictor in initial TAM studies (26), our finding suggests that for complex, skill-based technologies like VR, reducing ongoing operational friction (PE) may become equally or more important for continuance than reinforcing perceived benefits. This aligns with continuance studies emphasizing post-adoption effort expectancy (41).

### Theoretical implications

The findings offer two key theoretical contributions. First, they validate the utility of integrating constructs from adoption theories (UTAUT/TAM) within a continuance framework. By positioning CI as the dependent variable and demonstrating that SC, SI, and FC operate through the established mediators of PE and PU, this study provides a validated model for investigating sustained technology use in educational settings. This bridges a theoretical gap between research on initial acceptance and long-term engagement.

Second, the results delineate specific mediation pathways, offering a more nuanced understanding than a direct-effects model. The data indicate that system design and social influence primarily bolster perceptions of usefulness, while social influence and institutional support structures more strongly enhance perceptions of ease of use. Mapping these distinct routes clarifies how different types of interventions might target specific cognitive levers to foster continuance intention.

### Practical implications

The identified indirect pathways translate into actionable recommendations for different stakeholders. For Instructional Designers and Developers (Targeting SC→PU), the focus should extend beyond technical immersion to ensure that VR scenarios are pedagogically aligned and clinically relevant. Demonstrating straightforward utility for mastering specific competencies is paramount for fostering PU and, consequently, CI. For Educators and Administrators (Targeting SI→PE/PU), fostering a supportive community of practice is essential. Integrating the system into formal curricula, facilitating peer sharing of experiences, and showcasing instructor endorsements can amplify both its perceived usefulness and ease of use within the student community. For IT Support and Institutions (Targeting FC→PE), reducing post-adoption friction is crucial. Providing seamless access, reliable technical support, and intuitive guidance materials directly enhances PE, lowering the barrier to consistent use.

### Limitations and future research

This study’s inherent design limitations point to valuable future research directions. First, the cross-sectional data establish robust associative relationships but cannot definitively confirm causality or capture temporal dynamics in perception and intention. Longitudinal studies are needed to trace these evolutions. Second, while sampling experienced users was methodologically necessary, it limits the generalizability of findings to novice populations. Future work should examine how these mechanisms differ across stages of user experience. Third, the study focused on behavioral intention. Although intention is a strong predictor, research linking these perceptual constructs to actual sustained usage behavior and, separately, to objective learning outcomes, would provide a more complete assessment of VR system success.

## Conclusion

This study validated an integrated model to explain medical students’ continuance intention to use VR-based learning systems. The central finding is that SC, SI, and FC do not directly affect continuance intention but exert their influence fully through the mediators of PE and PU. Specifically, the analysis delineated two primary pathways: (1) SC and SI bolster CI by enhancing PU, and (2) SI and FC bolster CI by enhancing PE. Notably, PE emerged as the strongest direct driver of CI, underscoring the critical importance of minimizing post-adoption effort for sustained use.

These results bridge adoption and continuance theories, providing a validated framework for understanding sustained technology engagement in medical education. For practice, they offer clear, evidence-based guidance: instructional designers should focus on pedagogical relevance to enhance usefulness, educators should leverage social communities to reinforce both ease of use and usefulness, and institutions must invest in robust technical support to reduce usage barriers. Future longitudinal research is recommended to trace the evolution of these perceptions and link them to objective learning outcomes.

## Data Availability

The raw data supporting the conclusions of this article will be made available by the authors, without undue reservation.

## References

[1] Suliman M Zhang W Suluman R Sleiman K. Medical student’s acceptance of mobile learning: integrating TAM model with perceived reusability. *Educ Inf Technol.* (2025) 30:3621–44. 10.1007/s10639-024-12917-3

[2] Cheng Y. Investigating medical professionals’ continuance intention of the cloud-based e-learning system: an extension of expectation–confirmation model with flow theory. *J Enterp Inf Manag.* (2020) 34:1169–202. 10.1108/JEIM-12-2019-0401

[3] Hammouda S Maoua M Bouchahma M. The effectiveness of VR-based human anatomy simulation training for undergraduate medical students. *BMC Med Educ.* (2025) 25:816. 10.1186/s12909-025-07402-5 40452030 PMC12128225

[4] Einloft J Bedenbender S Michelsen M Meyer H Russ P Heidtmann A Structured exposure achieves high acceptance of immersive technology among medical students and educators. *Cyberpsychol Behav Soc Netw.* (2024) 27:363–71. 10.1089/cyber.2023.0297 38513055

[5] Mergen M Graf N Meyerheim M. Reviewing the current state of virtual reality integration in medical education - a scoping review. *BMC Med Educ.* (2024) 24:788. 10.1186/s12909-024-05777-5 39044186 PMC11267750

[6] Briz-Ponce L Pereira A Carvalho L Juanes-Méndez J García-Peñalvo F. Learning with mobile technologies – Students’ behavior. *Comput Hum Behav.* (2017) 72:612–20. 10.1016/j.chb.2016.05.027

[7] Noble S Saville J Foster LL. VR as a choice: what drives learners’ technology acceptance? *Int J Educ Technol High Educ.* (2022) 19:6. 10.1186/s41239-021-00310-w

[8] Van Raaij E Schepers J. The acceptance and use of a virtual learning environment in China. *Comput Educ.* (2008) 50:838–52. 10.1016/j.compedu.2006.09.001

[9] Garavand A Samadbeik M Nadri H Rahimi B Asadi H. Effective factors in adoption of mobile health applications between medical sciences students using the UTAUT model. *Methods Inf Med.* (2019) 58:131–9. 10.1055/s-0040-1701607 32170717

[10] Bhattacherjee A. Understanding information systems continuance: an expectation-confirmation model. *MIS Quarterly.* (2001) 25:351–70. 10.2307/3250921

[11] Lu X Gong D Zhang X Kim K. Continuance intention of older adults to adopt virtual reality application: insights from SEM and fsQCA analysis. *Geriatr Nurs.* (2025) 65:103527. 10.1016/j.gerinurse.2025.103527 40664136

[12] Wu C Chen C Huang K Chou Y. Determinants of Chatbot adoption among older adults: an extended TAM approach using PLS-SEM. *Inf Dev.* (2025) 41:656–74. 10.1177/02666669251315839

[13] Alfalah S Falah J Alfalah T Elfalah M Muhaidat N Falah OA. comparative study between a virtual reality heart anatomy system and traditional medical teaching modalities. *Virtual Real.* (2019) 23:229–34. 10.1007/s10055-018-0359-y

[14] Xu X Mangina E Campbell AG. HMD-based virtual and augmented reality in medical education: a systematic review. *Front Virtual Real.* (2021) 2:692103. 10.3389/frvir.2021.692103

[15] Mistry D Brock C Lindsey T. The present and future of virtual reality in medical education: a narrative review. *Cureus.* (2023) 15:e51124. 10.7759/cureus.51124 38274907 PMC10810257

[16] Radianti J Majchrzak T Fromm J Wohlgenannt I. A systematic review of immersive virtual reality applications for higher education: design elements, lessons learned, and research agenda. *Comput Educ.* (2020) 147:103778. 10.1016/j.compedu.2019.103778

[17] Zhao G Fan M Yuan Y Zhao F Huang H. The comparison of teaching efficiency between virtual reality and traditional education in medical education: a systematic review and meta-analysis. *Ann Transl Med.* (2021) 9:252–252. 10.21037/atm-20-2785 33708879 PMC7940910

[18] Barteit S Lanfermann L Bärnighausen T Neuhann F Beiersmann C. Augmented, mixed, and virtual reality-based head-mounted devices for medical education: systematic review. *JMIR Serious Games.* (2021) 9:e29080. 10.2196/29080 34255668 PMC8299342

[19] Zhao J Xu X Jiang H Ding Y. The effectiveness of virtual reality-based technology on anatomy teaching: a meta-analysis of randomized controlled studies. *BMC Med Educ.* (2020) 20:127. 10.1186/s12909-020-1994-z 32334594 PMC7183109

[20] Moro C Štromberga Z Raikos A Stirling A. The effectiveness of virtual and augmented reality in health sciences and medical anatomy. *Anat Sci Educ.* (2017) 10:549–59. 10.1002/ase.1696 28419750

[21] Aydin M Curran V White S Peña-Castillo L Meruvia-Pastor O. VR-NRP: a development study of a virtual reality simulation for training in the neonatal resuscitation program. *Digit Health.* (2025) 11:20552076251323989. 10.1177/20552076251323989 40103644 PMC11915296

[22] Jans C Bogossian F Andersen P Levett-Jones T. Examining the impact of virtual reality on clinical decision making – An integrative review. *Nurse Educ Today* (2023) 125:105767. 10.1016/j.nedt.2023.105767 36906980

[23] Bracq M Michinov E Jannin P. Virtual reality simulation in non-technical skills training for healthcare professionals: a systematic review. *Simul Healthc.* (2019) 14:188. 10.1097/SIH.0000000000000347 30601464

[24] Sattar M Palaniappan S Lokman A Hassan A Shah N Riaz Z. Effects of Virtual Reality training on medical students’ learning motivation and competency: medical students’ learning motivation & competency. *Pak J Med Sci.* (2019) 35:852–7. 10.12669/pjms.35.3.44 31258607 PMC6572943

[25] Junga A Schulze H Scherzer S Haetscher O Bozdere P Schmidle P Immersive learning in medical education: analyzing behavioral insights to shape the future of VR-based courses. *BMC Med Educ.* (2024) 24:1413. 10.1186/s12909-024-06337-7 39627755 PMC11616111

[26] Davis F. Perceived usefulness, perceived ease of use, and user acceptance of information technology. *MIS Quarterly.* (1989) 13:319–40. 10.2307/249008

[27] Venkatesh V Morris M Davis G Davis F. User acceptance of information technology: toward a unified view. *MIS Quarterly.* (2003) 27:425–78. 10.2307/30036540

[28] Kim S Lee K Hwang H Yoo S. Analysis of the factors influencing healthcare professionals’ adoption of mobile electronic medical record (EMR) using the unified theory of acceptance and use of technology (UTAUT) in a tertiary hospital. *BMC Med Inform Decis Mak.* (2015) 16:12. 10.1186/s12911-016-0249-8 26831123 PMC4736616

[29] Nascimento B Oliveira T Tam C. Wearable technology: what explains continuance intention in smartwatches? *J Retail Consum Serv.* (2018) 43:157–69. 10.1016/j.jretconser.2018.03.017

[30] Bai B Guo Z. Understanding users’ continuance usage behavior towards digital health information system driven by the digital revolution under COVID-19 context: an extended UTAUT model. *PRBM.* (2022) 15:2831–42. 10.2147/PRBM.S364275 36212806 PMC9532259

[31] Roca J Chiu C Martínez F. Understanding e-learning continuance intention: an extension of the technology acceptance model. *Int J Hum-Comput Stud.* (2006) 64:683–96. 10.1016/j.ijhcs.2006.01.003

[32] Nguyen T Pham YB. E-payment continuance intention: evidence from vietnam. *J Distrib Sci.* (2025) 23:13–22. 10.15722/JDS.23.07.202507.13

[33] Lutfi A. Factors influencing the continuance intention to use accounting information system in jordanian SMEs from the perspectives of UTAUT: top management support and self-efficacy as predictor factors. *Economies.* (2022) 10:75. 10.3390/economies10040075

[34] Zhao Y Bacao F. What factors determining customer continuingly using food delivery apps during 2019 novel coronavirus pandemic period? *Int J Hosp Manag.* (2020) 91:102683. 10.1016/j.ijhm.2020.102683 32929294 PMC7480677

[35] Cioc M Popa S Olariu A Popa C Nica C. Behavioral intentions to use energy efficiency smart solutions under the impact of social influence: an extended TAM approach. *Appl Sci-Basel.* (2023) 13:10241. 10.3390/app131810241

[36] Balkaya S Akkucuk U. Adoption and use of learning management systems in education: the role of playfulness and self-management. *Sustainability.* (2021) 13:1127. 10.3390/su13031127

[37] Fornell C Larcker D. Evaluating structural equation models with unobservable variables and measurement error. *J Mark Res.* (1981) 18:39–50. 10.1177/002224378101800104

[38] Bagozzi R Yi Y. On the evaluation of structural equation models. *JAMS.* (1988) 16:74–94. 10.1007/BF02723327

[39] Lim C Liew K Lim S Cheema M Sulaiman P Harith H Student acceptance towards AsepticTech VR: a teaching and learning tool for cell and tissue culture aseptic techniques. *Int J Educ Technol High Educ.* (2024) 21:36. 10.1186/s41239-024-00472-3

[40] Argan M Gurbuz B Dursun M Dinc H Tokay Argan M. Usage intention of ChatGPT for health in Turkey: an extended technology acceptance model. *Int J Hum-Comput Interact.* (2025) 41:9492–504. 10.1080/10447318.2024.2426045

[41] Thong J Hong S Tam K. The effects of post-adoption beliefs on the expectation-confirmation model for information technology continuance. *Int J Hum-Comput Stud.* (2006) 64:799–810. 10.1016/j.ijhcs.2006.05.001

[42] Ashfaq M Yun J Yu S Loureiro SMC. I, Chatbot: Modeling the determinants of users’ satisfaction and continuance intention of AI-powered service agents. *Telemat Inform.* (2020) 54:101473. 10.1016/j.tele.2020.101473

[43] Wu B Chen X. Continuance intention to use MOOCs: integrating the technology acceptance model (TAM) and task technology fit (TTF) model. *Comput Hum Behav.* (2017) 67:221–32. 10.1016/j.chb.2016.10.028

